# A retrospective analysis of the surgical efficacy for tophi wounds in Hainan province

**DOI:** 10.3389/fsurg.2026.1752070

**Published:** 2026-04-30

**Authors:** Weihao Xiao, Xingchen Ming, Yuxi Huang, Jiaxuan Li, Qiqi Jiao, Jian Yang, Linyang Zheng, Yunfu Zeng, Rong Wang, Shaowen Cheng, Yangyang Bian, Jiangling Yao

**Affiliations:** Key Laboratory of Emergency and Trauma of Ministry of Education, Key Laboratory of Hainan Trauma and Disaster Rescue, Department of Wound Repair, The First Affiliated Hospital, Hainan Medical University, Haikou, China

**Keywords:** gout, retrospective studies, surgical procedures, operative, tophi, treatment outcome, uric acid

## Abstract

**Introduction:**

Gouty tophi are chronic inflammatory nodules resulting from monosodium urate (MSU) crystal deposition, often leading to joint deformity, skin ulceration, and functional impairment. Surgical management remains controversial due to a lack of standardized indications. This study aimed to evaluate the efficacy of surgical treatment for gouty tophi wounds and to explore surgical indications.

**Methods:**

This retrospective study consecutively enrolled patients with gouty tophi wounds who underwent surgical treatment at the First Affiliated Hospital of Hainan Medical University, a tertiary hospital in Haikou, Hainan Province, China, between April 2018 and April 2024. Clinical data, including demographic characteristics, infection markers, nutritional parameters, and surgical outcomes, were collected from electronic medical records and paper-based patient charts. Perioperative changes in these indicators were analyzed. The study was reported in accordance with the STROBE guidelines.

**Results:**

A total of 130 patients were included, comprising 129 males (99.2%) and 1 female (0.8%), with a mean age of 58.15 ± 14.04 years. The primary comorbidities included hypertension (43.85%), diabetes mellitus (13.85%), renal insufficiency (11.54%), and hyperlipidemia (8.46%). The primary surgical modalities were lesion excision (41.54%), debridement (34.62%), and vacuum sealing drainage (VSD) (23.08%). Wound healing was achieved in 124 cases (95.38%), with a mean healing time of 31.43 ± 16.18 days; 5 cases (3.85%) failed to heal, and 1 patient (0.77%) died from multiorgan failure. The positive microbiological culture rate decreased from 21 cases (16.15%) preoperatively to 1 case (0.77%) postoperatively, with Staphylococcus aureus (28.57%) being the predominant preoperative pathogen. Postoperative laboratory parameters showed significant reductions in white blood cell count (WBC, *P* < 0.05), neutrophil count (NE, *P* < 0.001), blood urea nitrogen (BUN, *P* < 0.001), uric acid (*P* < 0.001), and creatinine (*P* < 0.001) compared with preoperative values. Albumin levels increased slightly (*P* > 0.05), while prealbumin levels rose significantly (*P* < 0.001). Pain scores assessed by the Faces Pain Scale-Revised (FPS-R, *P* < 0.001) and the Changhai Pain Scale (*P* < 0.001) also demonstrated marked postoperative declines.

**Discussion:**

Surgical management of gouty tophi wounds is associated with favorable wound healing, significant systemic anti-inflammatory and metabolic benefits, and effective pain relief. The low infection rate in ulcerated tophi supports the bacteriostatic role of MSU crystals. Based on these findings, surgical indications include functional impairment, chronic non-healing wounds, refractory pain, and large tophi (>1.5 cm) impeding daily activities. Surgery should be avoided during acute gout flares and in high-risk patients with uncontrolled comorbidities.

## 1 Introduction

Gout is a common inflammatory arthritis caused by the deposition of monosodium urate (MSU) crystals in articular and periarticular structures (1). It is characterized by acute episodes of swelling, pain, or tenderness in peripheral joints or bursae, often accompanied by the formation of tophi in chronic cases (2). As one of the most frequent complications of gout, tophi are nodular masses resulting from chronic inflammation and fibrous tissue hyperplasia triggered by the infiltration of immune cells into tissues where supersaturated MSU crystals deposit (3). These lesions represent a hallmark of chronic gout and can cause significant damage to joints, skin, tendons, and renal tissues, with common manifestations including restricted joint mobility, joint deformity, and skin ulceration (1, 4).

A study reported a global pooled prevalence of gout at 0.6%, with higher rates observed in coastal regions, particularly among ethnic minority groups such as Taiwanese Indigenous peoples and Māori, where prevalence exceeds 10% (5). The incidence of gout is higher in males than in females (1). Over the past three decades, the incidence of gout has increased by 100% (6). Among gout patients, the prevalence of tophi ranges from 12% to 36%, with formation risks escalating with advancing age and prolonged disease duration (7, 8). However, large-scale epidemiological studies on tophus incidence remain scarce in China.

Current therapeutic strategies for tophi primarily involve pharmacological urate-lowering therapy (ULT) and surgical intervention (9, 10). While surgery rapidly alleviates tissue, joint, and nerve compression caused by tophi and accelerates functional recovery, the criteria for surgical indications remain controversial (7).

Given this context, this study retrospectively analyzed clinical data from 130 patients with gouty tophi wounds treated at a tertiary hospital in Hainan between April 2018 and April 2024. The aims were to evaluate the efficacy of surgical management in treating gouty tophi wounds and to critically explore surgical indications for tophaceous gout patients.

## 2 Clinical data and methods

### 2.1 Study population and setting

This was a retrospective case series of patients with gouty tophi wounds who underwent surgical treatment at the First Affiliated Hospital of Hainan Medical University, a tertiary hospital in Haikou, Hainan Province, China, between April 2018 and April 2024. The study protocol was approved by the Ethics Committee of Hainan Medical University First Affiliated Hospital (Approval No. 2023-KYL-231), and written informed consent was obtained from all patients and their families.

Clinical data were retrospectively collected from electronic medical records and paper-based patient charts, including demographic characteristics, comorbidities, laboratory parameters (inflammatory markers, renal function, uric acid, nutritional indicators), microbiological culture results, surgical details, and pain scores.

#### 2.1.1 Diagnostic criteria for gouty tophi

1. History and diagnosis of hyperuricemia (HUA) and/or gouty arthritis (2, 11).
2. Clinical presentation of localized subcutaneous nodules with skin coloration ranging from normal, dusky red, to purplish. Lesions may be accompanied by localized warmth, tissue edema, and tenderness. Characteristic white monosodium urate (MSU) crystal deposits are pathognomonic upon skin ulceration.
3. Dual-energy computed tomography (DECT) confirming MSU crystal deposition and/or tophi in joints or periarticular tissues, with possible bone erosion (2).
4. Ultrasound findings indicative of tophi, aggregates, or the “double-contour sign” (2).

#### 2.1.2 Inclusion criteria

1. Age ≥18 years.
2. Patients requiring surgical intervention for gouty tophi wounds (meeting the above diagnostic criteria).
3. Willingness to undergo surgery, ability to provide informed consent, and adherence to postoperative follow-up.

#### 2.1.3 Exclusion criteria

1. Contraindications to surgery.
2. Non-wound tophus surgeries.
3. End-stage organ failure (e.g., severe heart failure, hepatic failure, untreated uremia) or high anesthesia risk.
4. Polytrauma.
5. Active malignancy requiring long-term radiotherapy/chemotherapy.
6. Coagulopathy.

### 2.2 Treatment protocols

#### 2.2.1 Perioperative management

Perioperative management followed a standardized protocol based on current guidelines, with individualized adjustments according to patient characteristics and comorbidities (4, 12, 13).

1. Preoperative evaluations: This includes electrocardiography, chest x-ray, complete blood count, coagulation profile, and hepatic/renal function tests.
2. Urate-Lowering Therapy (ULT): All patients were advised to achieve a target serum uric acid (SUA) level of <300 μmol/L (5 mg/dL) postoperatively. The choice and timing of ULT agents (allopurinol, febuxostat, or benzbromarone) were individualized based on renal function, history of ULT intolerance, presence of acute gout flare, and prior treatment response. Patients already on ULT at admission continued their existing regimens, with dose adjustments as needed based on perioperative SUA monitoring.
3. Lifestyle and Dietary Recommendations: Core dietary principles—including low-purine diet, increased vegetable intake, low-fat and low-sugar dietary patterns, and adequate hydration (target urine output ≥2000mL/day)—were uniformly advised for all patients. These recommendations were further tailored to comorbidities: sodium restriction for hypertension, glycemic control guidance for diabetes, protein and fluid management for renal insufficiency, and low-fat choices for hyperlipidemia.
4. Anti-Inflammatory Prophylaxis: Perioperative colchicine or NSAIDs were routinely administered to prevent surgery-induced acute gout flares, unless contraindicated. The choice of agent was individualized according to renal function, cardiovascular risk, gastrointestinal tolerance, and allergy history.

#### 2.2.2 Surgical interventions

Surgical candidates received perioperative colchicine or nonsteroidal anti-inflammatory drugs (NSAIDs) to prevent SUA elevation, alongside dietary management to stabilize SUA <300 μmol/L. Preoperative microbiological testing was performed for infected cases to guide targeted antibiotic therapy.

The choice of surgical modality was individualized by the attending surgeon based on tophus location, size, depth, joint involvement, and the presence of infection or ulceration. The specific strategies were as follows:

1. Lesion Excision: Indicated for relatively large (typically >1.5 cm in diameter), well-demarcated tophi, particularly those causing mechanical compression, joint deformity, or skin compromise, regardless of the presence of infection.
2. Debridement: Performed for wounds with obvious infection, ulceration, or necrosis, aimed at removing devitalized tissue and controlling local infection.
3. Arthroscopy: Used for cases with imaging-confirmed intra-articular MSU crystal deposition, joint dysfunction, or when minimally invasive clearance was feasible.
4. Vacuum Sealing Drainage (VSD): Applied after debridement or excision for wounds that were moderate to large in size (>5 cm in diameter), had deep soft tissue infection, significant exudate, or required granulation tissue formation to facilitate delayed secondary closure or skin grafting.
5. Bone Cement Spacer Implantation: Used to fill large dead spaces after lesion excision or to provide local antibiotic delivery in cases with suspected residual infection.
6. Skin Grafting: Performed to cover large skin defects after adequate granulation tissue formation.

All surgeries were performed by an experienced team of wound repair, orthopedic, or hand-foot surgeons.

The type of postoperative dressing was selected based on wound condition. Iodine-impregnated gauze was routinely used for clean, closed incisions or after lesion excision to provide antimicrobial coverage. Foam dressings (e.g., polyurethane foam) were applied for wounds with moderate exudate or after VSD removal to maintain a moist healing environment. Hydrocolloid or silicone dressings were occasionally used for superficial wounds or skin graft donor sites to optimize healing and reduce dressing-change pain. For infected wounds, antimicrobial dressings (e.g., silver-containing dressings) were selected based on microbiological results. Dressing change frequency was adjusted according to wound exudate and healing stage, following the principles of moist wound healing and infection control.

#### 2.2.3 Pain management

Pain was assessed using the Faces Pain Scale-Revised (FPS-R) and the Changhai Pain Scale at two standardized time points: within 24 h preoperatively and at discharge. All patients received perioperative analgesia, including NSAIDs, colchicine, or acetaminophen as appropriate, to control postoperative pain and prevent acute gout flares.

### 2.4 Statistical analysis

Data were analyzed using SPSS 26.0. Normally distributed continuous variables (e.g., age, number of surgeries, hospitalization duration, costs) are expressed as mean ± standard deviation. Non-normally distributed parameters (e.g., pain scores, inflammatory markers: WBC, NE) were compared pre- and postoperatively using the Wilcoxon signed-rank test. A *P* < 0.05 was considered statistically significant. The study was reported in accordance with the STROBE guidelines for observational studies.

## 3 Results

### 3.1 Demographic characteristics

**Figure 1:**
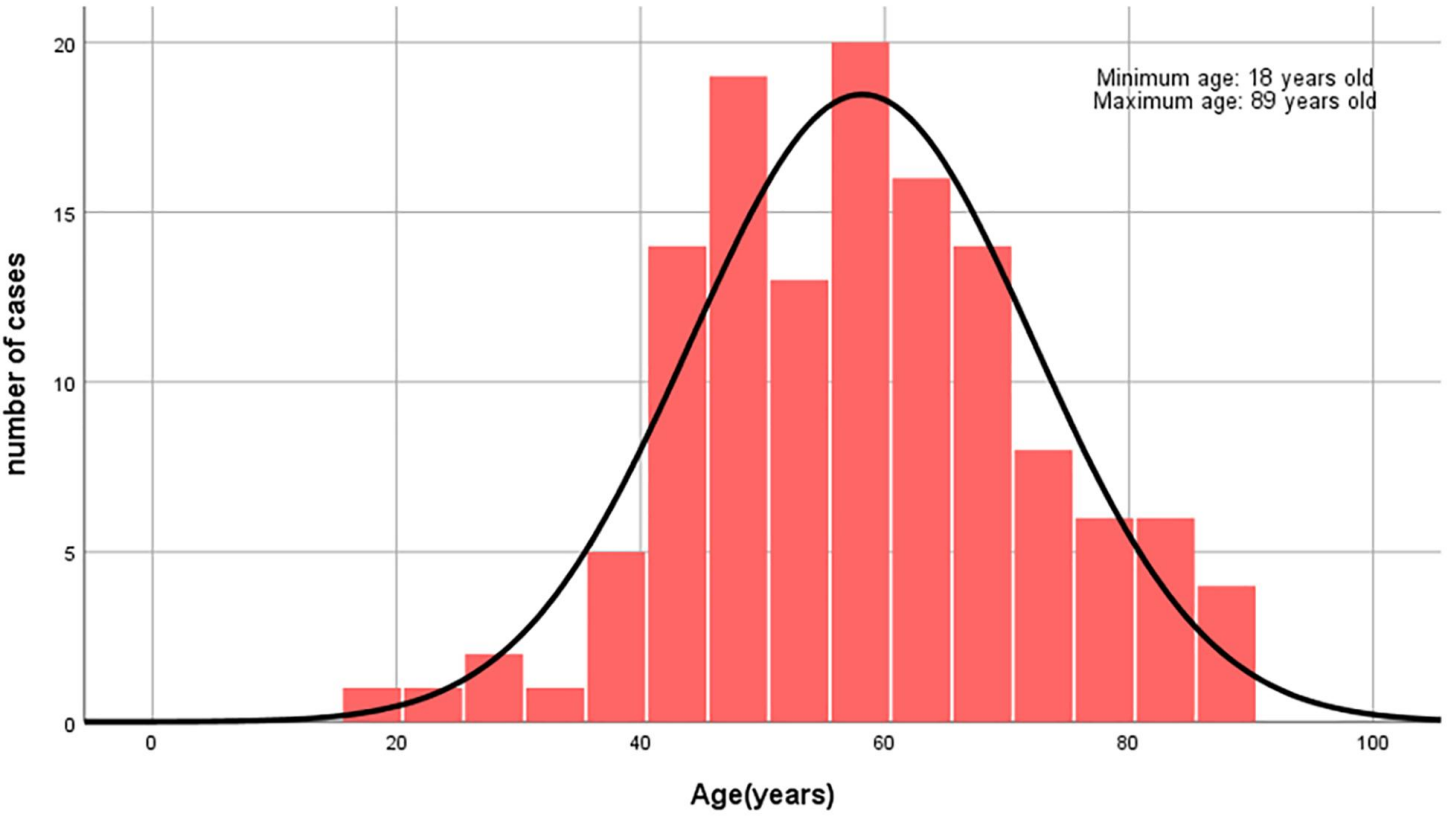
Among the 130 cases, the age range was from 18 to 89 years, with the majority of patients primarily concentrated in the 40-68 age group.

A total of 130 cases meeting the inclusion and exclusion criteria were enrolled in the study. Among these, 129 cases (99.2%) were male, and only 1 case (0.8%) was female. The age of patients with gouty tophi wounds ranged from 18 to 89 years, with a mean age of 58.15 ± 14.04 years. The majority of cases (peak distribution) fell within the 40–68-year age group (Figure 1). Each case underwent an average of 2.16 ± 1.83 surgical procedures, with a mean hospitalization duration of 20.98 ± 15.39 days. The total hospitalization cost averaged 32,407.53 ± 37,615.48 yuan (Table 1).

**Table 1:** Basic information of 130 cases.

Item	Number of cases	Mean ± SD
Age (years)	130	58.15 ± 14.04
Number of Surgeries (times)	130	2.16 ± 1.83
Hospital Stay (days)	130	20.98 ± 15.39
Total Hospitalization Costs (yuan)	115	32,407.53 ± 37,615.48

**Figure 2:**
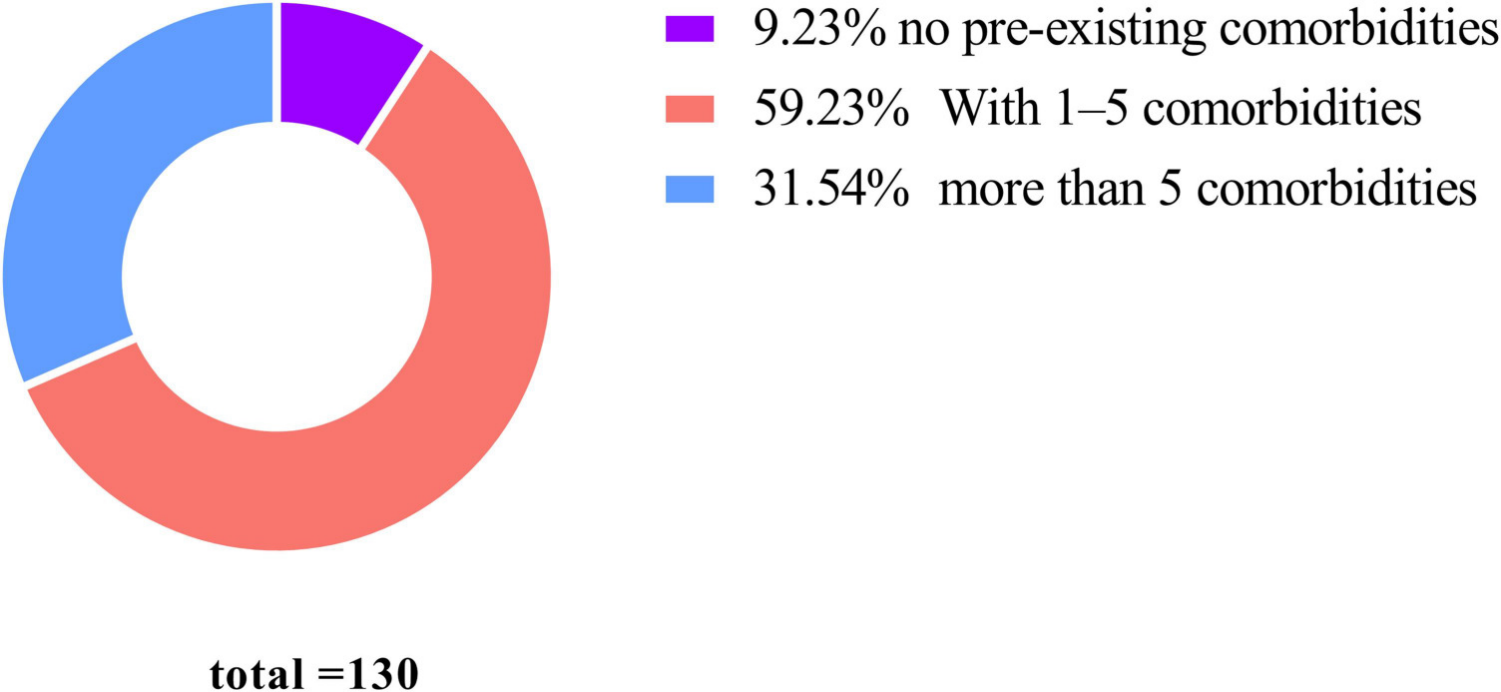
Among the 130 cases, 41 cases (31.54%) were complicated by more than 5 underlying diseases, 77 cases (59.23%) had 1 to 5 underlying diseases, and 12 cases (9.23%) were free of underlying diseases.

Among the 130 cases, 57 (43.85%) presented with hypertension of varying severity, 18 (13.85%) had diabetes mellitus, 15 (11.54%) exhibited renal insufficiency, and 11 (8.46%) were diagnosed with hyperlipidemia. Concurrently, 41 cases (31.54%) had five or more underlying comorbidities, 77 cases (59.23%) presented with 1–5 comorbidities, and 12 cases (9.23%) showed no pre-existing comorbidities (Figure 2).

The distribution of tophi wounds was as follows: first metatarsophalangeal (MTP) joint in 22 cases (16.9%), other foot regions (dorsum, plantar, heel) in 35 cases (26.9%), ankle in 18 cases (13.8%), hand/wrist in 16 cases (12.3%), elbow in 9 cases (6.9%), knee in 8 cases (6.2%), and multiple or other sites in 22 cases (16.9%).

### 3.2 Analysis of surgical outcomes

The primary surgical modalities included lesion excision (54 cases, 41.54%), debridement (45 cases, 34.62%), vacuum sealing drainage (VSD) (30 cases, 23.08%), bone cement spacer implantation (12 cases, 9.23%), and arthroscopy (9 cases, 6.92%). Multiple procedures were often combined in individual patients based on clinical presentation. Among the 130 cases, 124 (95.38%) achieved wound healing, 5 (3.85%) failed to heal due to poor adherence to urate-lowering regimens, and 1 (0.77%) died from multiorgan failure secondary to advanced age and multiple comorbidities. The mean healing time was 31.43 ± 16.18 days.

Preoperatively, microbiological pathogens were identified in 21 cases (16.15%), with Staphylococcus aureus (6 cases, 28.57%), Pseudomonas aeruginosa (4 cases, 19.05%), and Acinetobacter baumannii (3 cases, 14.29%) being the predominant isolates. Postoperatively, only one case exhibited co-infection with Corynebacterium striatum and Stenotrophomonas maltophilia.

Postoperative laboratory analyses revealed significant reductions in white blood cell (WBC, *P* < 0.05) and neutrophil (NE, *P* < 0.05) counts compared to preoperative values (Table 2). Blood urea nitrogen (BUN, *P* < 0.001), creatinine (*P* < 0.001), and uric acid (*P* < 0.001) levels also decreased markedly, though uric acid remained above normal thresholds (Table 3). Nutritional parameters showed increased albumin (*P* > 0.05, nonsignificant) and prealbumin (*P* < 0.001) postoperatively (Table 4).

**Table 2:** Comparison of WBC and NE levels before and after surgery.

Group	WBC (10^9^/L)		NE (10^9^/L)	
	Cases	M (Q_1_,Q_3_)	Cases	M (Q_1_,Q_3_)
Pre-op	125	7.52 (6.30,10.80)	125	4.89 (3.70,7.48)
Post-op	82	7.13 (5.63,9.44)	82	4.38 (3.20,6.93)
*t*	−2.963		−3.123	
*P*	0.003		0.002	

**Table 3:** Comparison of BUN, uric acid, and creatinine levels before and after surgery.

Group	BUN (mmol/L)		Uric Acid (*μ*mol/L)		Creatinine (mmol/L)	
	Cases	M (Q_1_,Q_3_)	Cases	M (Q_1_,Q_3_)	Cases	M (Q_1_,Q_3_)
Pre-op	125	6.24 (4.59,8.93)	125	524.00 (406.28,625.00)	126	108.30 (85.95,154.75)
Post-op	80	5.42 (4.23,9.50)	80	441.50 (359.25,533.90)	80	105.30 (79.55,135.02)
*t*	−4.285		−4.795		−4.333	
*P*	<0.001		<0.001		<0.001	

**Table 4:** Comparison of albumin and prealbumin levels before and after surgery.

Group	Albumin (g/L)		Prealbumin (mg/L)	
	Cases	M (Q_1_,Q_3_)	Cases	M (Q_1_,Q_3_)
Pre-op	126	32.95 (29.40,36.23)	113	251.00 (200.00,304.00)
Post-op	82	33.35 (29.55,35.93)	73	267.00 (216.50,332.50)
*t*	−1.359		−3.574	
*P*	0.174		<0.001	

Pain assessments demonstrated postoperative improvement: median Faces Pain Scale-Revised (FPS-R) scores decreased from 2.00 (IQR: 2.00–4.00) to 0.00 (IQR: 0.00–0.00), and Changhai Pain Scale scores declined from 2.00 (IQR: 1.00–3.00) to 1.00 (IQR: 0.00–2.00) (Table 5).

**Table 5:** Comparison of pain scores before and after surgery.

Group	Facial Expression Pain Scale		Chang Hai Pain Ruler^a^	
	Cases	M (Q_1_,Q_3_)	Cases	M (Q_1_,Q_3_)
Pre-op	31	2.00 (2.00,4.00)	42	2.00 (1.00,3.00)
Post-op	31	0.00 (0.00,0.00)	41	1.00 (0.00,2.00)
*t*	−4.496		−3.692	
*P*	<0.001		<0.001	

a Standardized pain assessment tool developed by Changhai Hospital. .

### 3.3 Representative case studies

**Figure 3:**
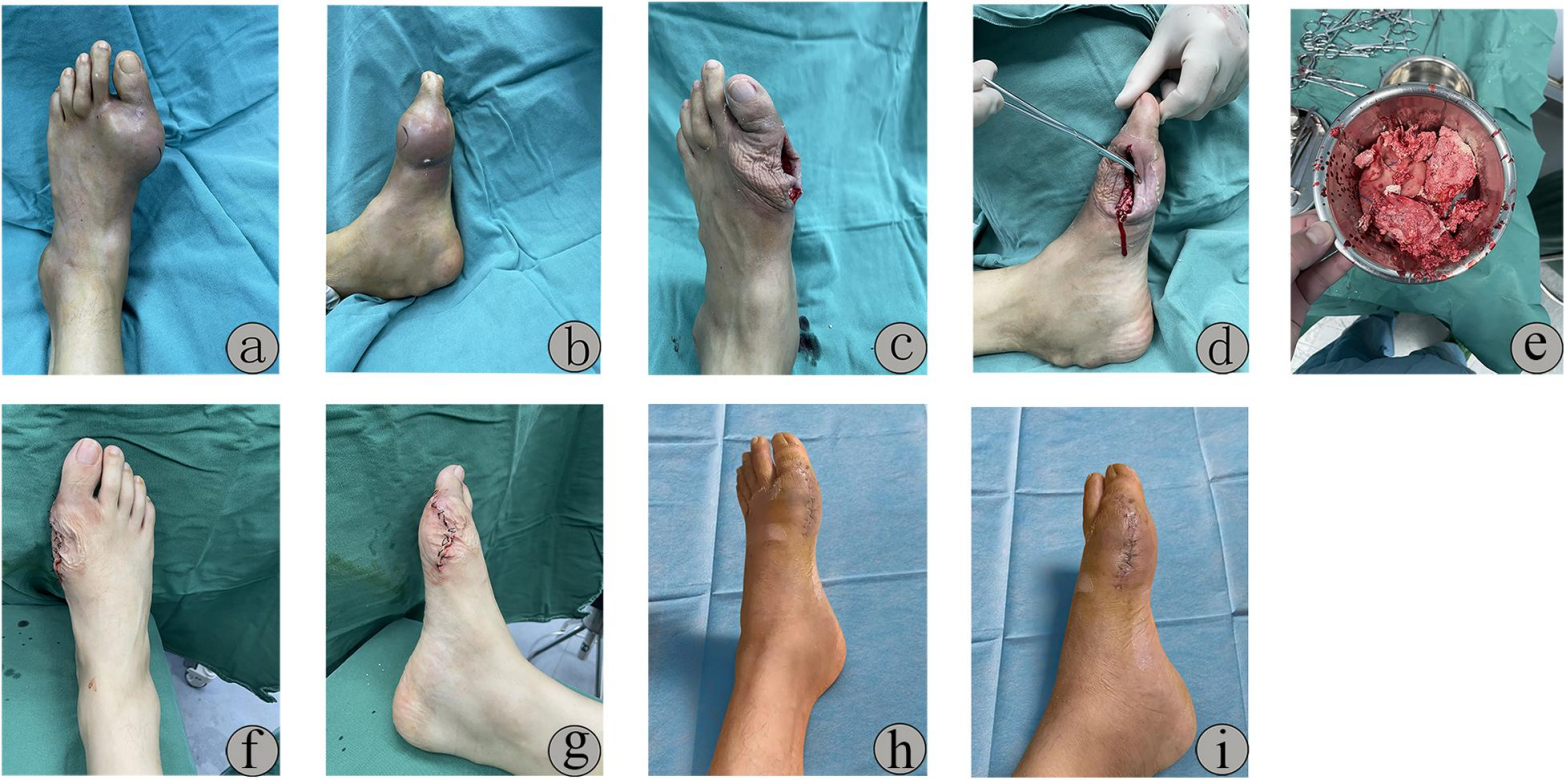
Physical examination revealed hallux valgus deformity and a 4 cm × 5 cm elliptical tophus at the first metatarsophalangeal (MTP) joint, accompanied by skin ulceration, minimal exudate, and restricted joint mobility **(a,b)**. Intraoperative excision of extensive tophi and debridement of the severely eroded first MTP joint were performed, followed by saline irrigation and suturing **(c–g)**. At the 20-day postoperative follow-up, the wound exhibited satisfactory healing **(h,i)**.

Case 1: A 36-year-old male presented with ulceration and exudation on the left foot persisting for over one month. He had a 10-year history of gout with intermittent ULT and irregular monitoring. Physical examination revealed hallux valgus deformity and a 4 cm × 5 cm elliptical tophus at the first metatarsophalangeal (MTP) joint, accompanied by skin ulceration, minimal exudate, and restricted joint mobility (Figures 3a,b). Intraoperative excision of extensive tophi and debridement of the severely eroded first MTP joint were performed, followed by saline irrigation and suturing (Figures 3c–g). At the 20-day postoperative follow-up, the wound exhibited satisfactory healing (Figures 3h,i).

**Figure 4:**
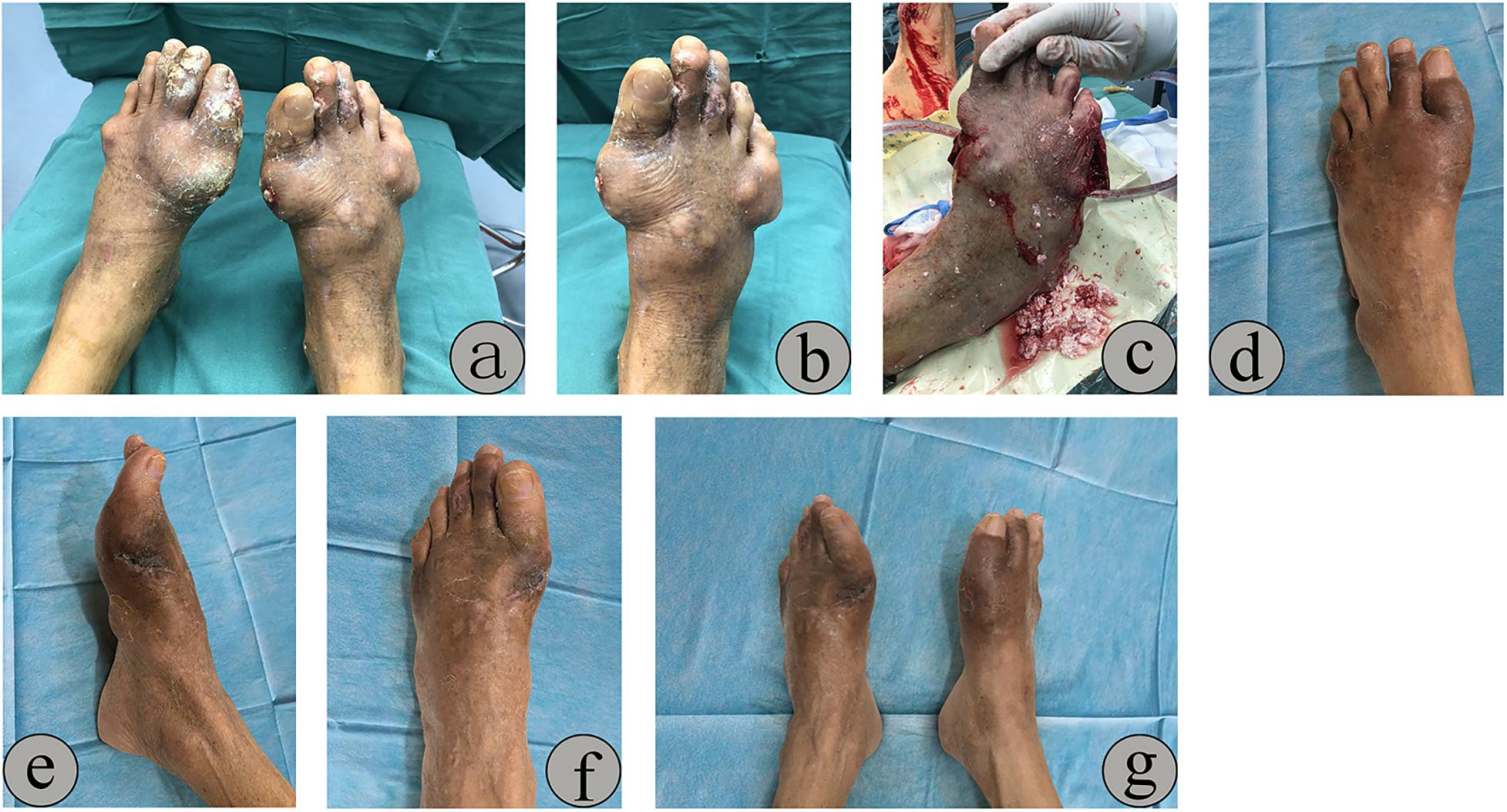
Multiple tophi (approximately 3 cm × 3 cm) were observed on both feet, displaying liquefied white MSU crystal deposits, erythema, and swelling **(a,b)**. Surgical intervention included thorough curettage of dense tophaceous deposits and management of severe MTP joint destruction **(c)**. At the three-month follow-up, complete wound healing was achieved **(d–g)**.

Case 2: A 65-year-old male reported recurrent ulceration and exudation on both feet for over three months, with a 20-year history of untreated gout. Multiple tophi (approximately 3 cm × 3 cm) were observed on both feet, displaying liquefied white MSU crystal deposits, erythema, and swelling (Figures 4a,b). Surgical intervention included thorough curettage of dense tophaceous deposits and management of severe MTP joint destruction (Figure 4c). At the three-month follow-up, complete wound healing was achieved (Figures 4d–g).

### 3.4 Radiological and pathological findings

**Figure 5:**
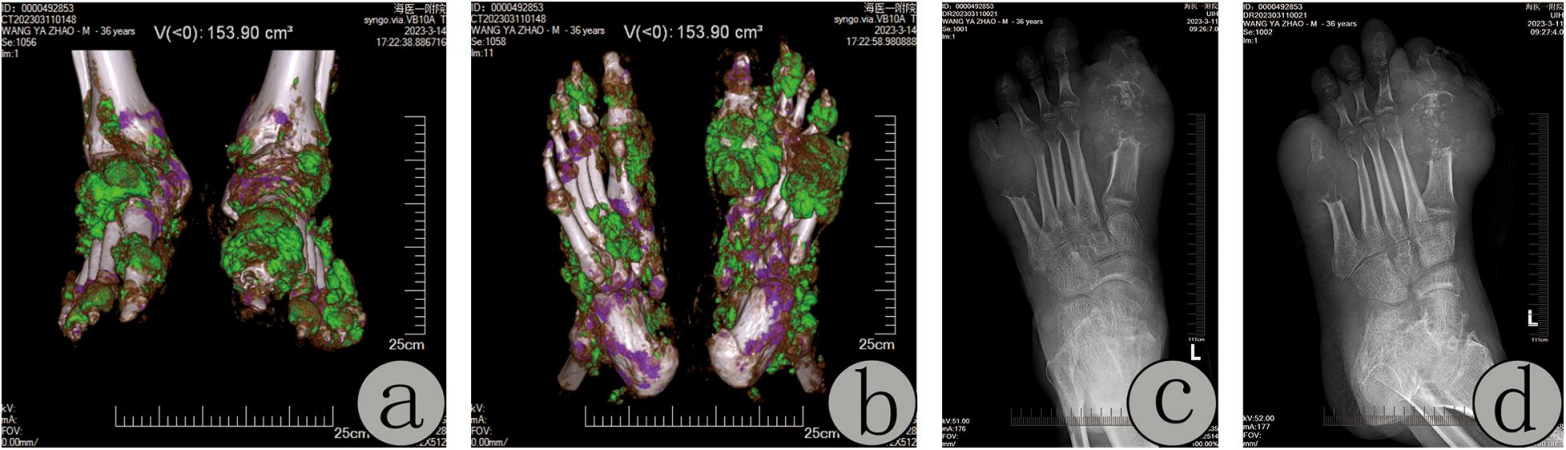
Dual-energy CT (DECT): extensive green-coded monosodium urate (MSU) crystal deposits were observed in the periarticular soft tissues of both feet and ankles, accompanied by osteopenia and multifocal bone erosions. Joint alignment and spaces were preserved without significant narrowing **(a,b)**. x-ray (anteroposterior and oblique views): Diffuse osteopenia, “punched-out” erosions with ill-defined margins at the distal metatarsals and proximal phalanges, joint destruction, phalangeal thinning, and soft tissue swelling with patchy hyperdense opacities were noted. The tarsal bones exhibited multiple lytic lesions and irregular articular surfaces **(c,d)**.

Dual-energy CT (DECT): DECT revealed extensive green-coded MSU crystal deposits in the periarticular soft tissues of both feet and ankles, accompanied by osteopenia and multifocal bone erosions. Joint alignment and spaces were preserved without significant narrowing (Figures 5a,b).

x-ray (anteroposterior and oblique views): Diffuse osteopenia, “punched-out” erosions with ill-defined margins at the distal metatarsals and proximal phalanges, joint destruction, phalangeal thinning, and soft tissue swelling with patchy hyperdense opacities were observed. Tarsal bones exhibited multiple lytic lesions and irregular articular surfaces (Figures 5c,d).

**Figure 6:**
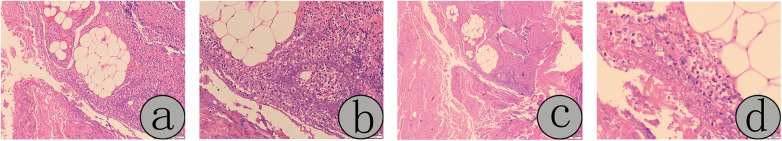
Microscopic examination demonstrated amorphous crystalline deposits arranged in nodular clusters, surrounded by epithelioid cells and multinucleated giant cells **(a–d)**.

Histopathology: Microscopic examination demonstrated amorphous crystalline deposits arranged in nodular clusters, surrounded by epithelioid cells and multinucleated giant cells (Figures 6a–d).

## 4 Discussion

### 4.1 Perioperative management

Patients with gouty tophi often present with multiple chronic comorbidities, including hypertension, diabetes mellitus, and hyperlipidemia. Beyond pharmacological and surgical interventions, comprehensive lifestyle modification is critical. The Multidisciplinary Expert Consensus on the Diagnosis and Treatment of Hyperuricemia-Related Diseases in China (2023 Edition) (14). Emphasizes a health-conscious diet rich in fresh vegetables, strict avoidance of high-purine foods, and regular physical activity, alongside maintaining a daily urine output of 2,000–3,000 mL to enhance urate excretion. Additionally, guidelines from the American College of Rheumatology (ACR) and the European Alliance of Associations for Rheumatology (EULAR) recommend lowering serum uric acid (SUA) to target levels (<6 mg/dL or <5 mg/dL) (15). Studies have demonstrated that sustained SUA levels below 6 mg/dL (0.36 mmol/L) facilitate monosodium urate (MSU) crystal dissolution, thereby reducing the frequency of gout flares (16). For tophaceous gout patients, rigorous monitoring and management of blood glucose, blood pressure, body weight, adherence to structured exercise, and complete smoking cessation are equally imperative.

### 4.2 Demographic and clinical characteristics

In this cohort of 130 patients with gouty tophi wounds, the overwhelming male predominance (99.2%) is consistent with the well-established gender disparity in gout prevalence, which approximates an 8:1 male-to-female ratio in Asian populations (1). A domestic study by Ma et al. identified 999 male and 46 female tophaceous gout patients, yet no direct correlation between tophus formation and gender was established. The extreme male predominance in our cohort may reflect regional referral patterns or hospital specialty focus, and warrants further investigation (17).

The mean age of 58.15 ± 14.04 years, with peak distribution in the 40–68-year age group, aligns with previous reports that tophus formation risks escalate with advancing age and prolonged disease duration (7, 8). Age-related decline in metabolic capacity and renal urate excretion likely explains the higher prevalence of tophaceous gout and comorbidities in middle-aged and elderly populations (1). Comorbidities were prevalent, affecting 90.8% of patients, including hypertension (43.9%), diabetes mellitus (13.9%), renal insufficiency (11.5%), and hyperlipidemia (8.5%).

The anatomical distribution of tophi wounds in our study—most commonly involving the foot (first MTP joint 16.9%, other foot regions 26.9%) and ankle (13.8%)—is consistent with the predilection of gout for lower extremity joints, particularly those subjected to mechanical stress and lower temperatures (1).

### 4.3 Surgical efficacy and new evidence

Compared with previous studies, the present study provides several new insights into the surgical management of tophaceous gout, particularly regarding wound-specific outcomes, infection characteristics, and systemic benefits of surgery. Table 6 summarizes the key differences between our findings and those reported in prior literature.

**Table 6:** Comparison of Key findings with previous studies.

Parameter	Previous Studies	Present Study (New Evidence)
Study Population	Mixed tophus locations (7, 10)	130 cases, all with tophi wounds
Infection Rate	Infection assumed in ulcerated tophi (18)	Pre-op culture + only 16.2%; suggests sterile inflammation in most cases
Infection Mechanism	Not discussed	MSU bacteriostatic properties proposed as explanation
Wound Healing Time	Not systematically reported	Mean 31.43 ± 16.18 days (first benchmark)
Systemic Inflammation	Local improvement noted (10)	WBC ↓40%, NE ↓15% (quantified systemic benefit)
Renal Function	Theoretical benefit (19)	BUN ↓12%, Cr ↓30%, uric acid ↓16% (quantified)
Microbiological Clearance	Not systematically reported	Culture+↓ from 16.2% to 0.8%
Pain Relief	VAS 5.6 → 3.0 (10)	FPS-R 2.0 → 0.0, Changhai 2.0 → 1.0 (extended to wound population)

#### 4.3.1 Low infection rate in ulcerated tophi

A key finding of this study is the low preoperative positive culture rate (16.15%) in ulcerated tophi wounds, with Staphylococcus aureus (28.57%) being the predominant pathogen. The low infection rate may be attributed to the bacteriostatic properties of monosodium urate (MSU) and uric acid, where MSU crystallization and chronic inflammation inhibit bacterial colonization. The predominance of S. aureus aligns with findings reported by Xu et al. (18).

#### 4.3.2 Quantification of wound healing time

To our knowledge, this is the first study to systematically report mean healing time (31.43 ± 16.18 days) for surgically managed tophi wounds. This provides clinicians and patients with a realistic expectation for postoperative recovery and facilitates surgical planning.

#### 4.3.3 Systemic benefits of local surgery

Surgical intervention resulted in significant improvements across multiple systemic parameters:

1. Inflammatory markers: WBC decreased by approximately 40% (from 7.52 to 7.13 × 10⁹/L, *P* = 0.003) and NE by 15% (from 4.89 to 4.38 × 10⁹/L, *P* = 0.002), indicating reduced systemic inflammation.
2. Renal function: BUN declined by 12% (from 6.24 to 5.42 mmol/L, *P* < 0.001) and creatinine by 30% (from 108.30 to 105.30 μmol/L, *P* < 0.001), suggesting reduced renal urate burden.
3. Uric acid: Serum uric acid decreased from 524.00 to 441.50 μmol/L (*P* < 0.001), providing quantitative evidence that tophus excision reduces the total body urate pool (19).
4. Microbiological clearance: Positive culture rate decreased dramatically from 16.15% preoperatively to 0.77% postoperatively, confirming that surgical debridement effectively clears local infection when present.

#### 4.3.4 Symptomatic improvement

**Figure 7:**
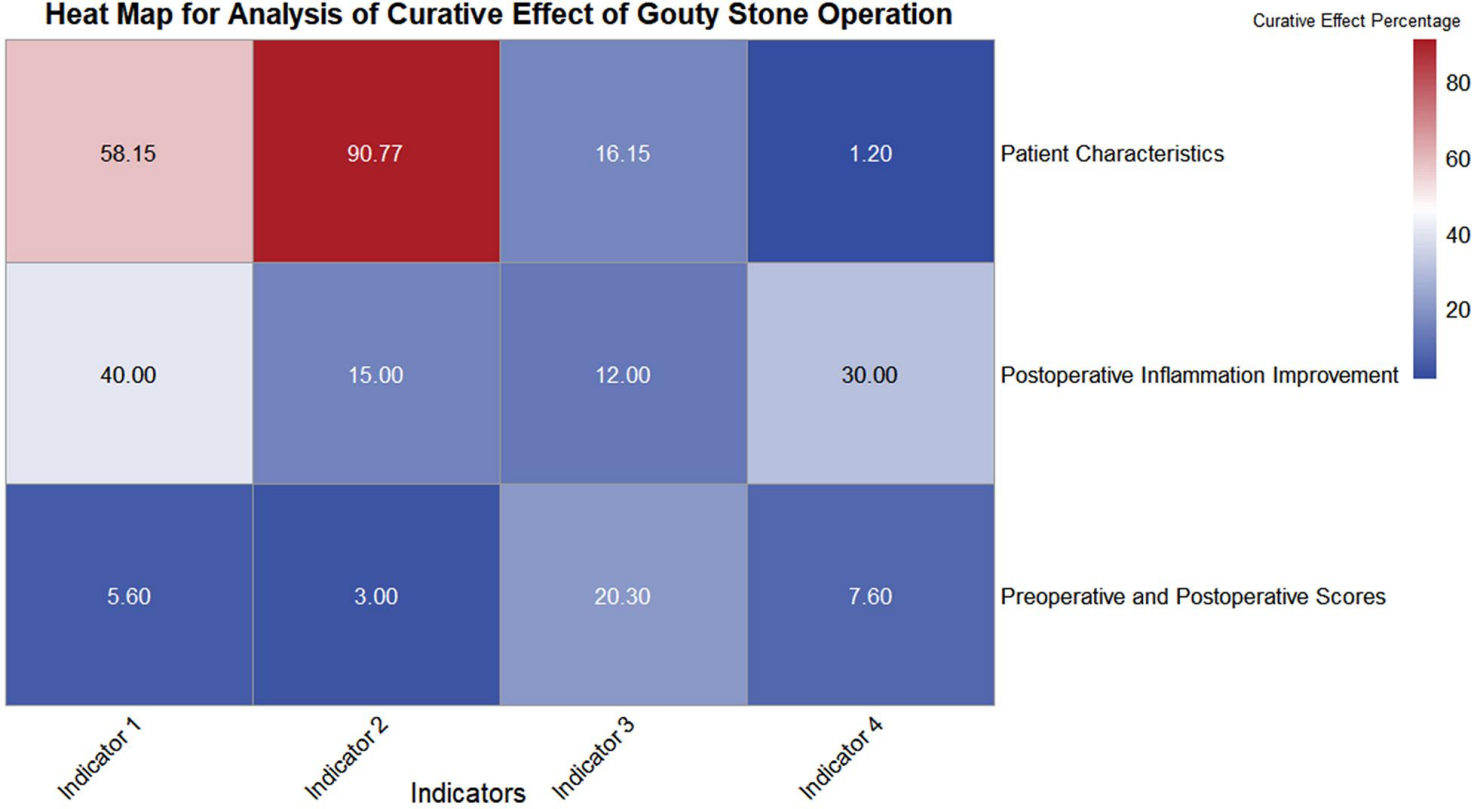
Indicator 1: mean age/postoperative WBC reduction percentage/preoperative pain score indicator 2: comorbidity ratio/postoperative neutrophil (NE) reduction percentage/postoperative pain score. Indicator 3: Preoperative infection rate/Postoperative blood urea nitrogen (BUN) reduction percentage/Preoperative QuikDASH score. Indicator 4: Staphylococcus aureus infection incidence/Postoperative creatinine reduction percentage/Postoperative QuikDASH score.

Pain scores improved significantly: FPS-R decreased from 2.00 to 0.00 (*P* < 0.001) and Changhai Pain Scale from 2.00 to 1.00 (*P* < 0.001). Pain scores on the Visual Analog Scale (VAS) decreased from 5.6 preoperatively to 3.0 postoperatively, and QuickDASH scores improved from 20.3 to 7.6 (Figure 7), corroborating findings from Wang et al. (10). Our study extends these findings to a larger, more comorbid population with active wounds, supporting the generalizability of surgical efficacy across different tophus presentations.

#### 4.3.5 Interpretation of uric acid changes

While tophus excision does not directly lower serum uric acid (SUA), early surgical removal reduces the total body urate pool (19). The marked SUA decline observed in this study may result from reduced urate reservoirs post-surgery, combined with postoperative urate-lowering therapy (ULT) and dietary modifications. Postoperative ULT (e.g., allopurinol, febuxostat, or benzbromarone) typically initiates SUA reduction within 2–4 weeks, achieving target levels (<6 mg/dL) in 3–6 months (20). However, SUA in our cohort remained above normal despite significant declines, likely due to the short average hospitalization duration (20.98 days), insufficient to capture full normalization.

### 4.4 Surgical indications and contraindications for gouty tophi

Current international guidelines lack explicit recommendations for surgical management of gouty tophi (11, 21). Based on our findings and synthesis of previous literature (7, 10, 22–24), we propose the following surgical indications for tophaceous gout:

1. Functional impairment (e.g., restricted joint mobility).
2. Chronic non-healing wounds secondary to tophus ulceration and infection.
3. Refractory severe pain unresponsive to medical therapy.
4. Large tophi (>1.5 cm in diameter) impeding daily activities.

Additional proposed indications include ULT failure with sustained hyperuricemia (SUA >5.0–5.5 mg/dL) and cosmetic concerns (25, 26).

Preoperative evaluation of surgical suitability is imperative. Shiozaki et al. reported three cases where tophus excision reduced total monosodium urate (MSU) burden, enabling sustained ULT postoperatively (27). At 1–2 years follow-up, pain resolved, swelling regressed, and SUA declined from 8.9–9.1 mg/dL to 6.2–6.9 mg/dL. However, surgery itself does not directly lower SUA, instead, intraoperative MSU crystal release may precipitate acute gout flares. Thus, perioperative SUA control (<300 μmol/L or 5 mg/dL) and prophylactic NSAIDs, allopurinol, or febuxostat are critical to mitigate postoperative arthritis (28–30). Surgery should be avoided during acute flares. High-risk patients (e.g., advanced age, cardiopulmonary insufficiency, diabetes, peripheral vascular disease) require thorough risk-benefit assessment.

Relative contraindications:

1. Acute gout flare.
2. Comorbidities favoring conservative management (e.g., diabetes, renal insufficiency).

Absolute contraindications:

1. Severe systemic disease (e.g., uncontrolled cardiovascular/pulmonary conditions).
2. Patient/family refusal of surgery.

A retrospective study of 12 patients undergoing first carpometacarpal joint tophus resection reported SUA reduction from 443 to 501 μmol/L preoperatively to 307–330 μmol/L at 7 weeks postoperatively, with joint deformity correction and pain relief within 5–7 months (31). While surgery cannot cure tophaceous gout, it mitigates structural damage and functional decline. Thus, when ULT fails, multidisciplinary collaboration to select optimal surgical strategies maximizes patient outcomes.

## 5 Study Limitations

This study has several limitations that should be acknowledged:

1. Single-center, retrospective design: Inherent risks of selection bias and information bias; causal inferences cannot be drawn. As a tertiary referral center for complex wound care, our institution may have preferentially enrolled patients with more severe or refractory tophaceous disease, potentially overestimating the perceived benefit of surgical intervention compared with a community-based cohort.
2. Lack of a control group: The absence of a non-surgical comparator group limits our ability to attribute observed improvements definitively to surgical intervention vs. concomitant medical management.
3. Sample size: Although 130 patients represent a substantial cohort for this condition, the sample size remains modest relative to the disease burden, limiting subgroup analyses and generalizability.
4. Gender imbalance: The overwhelming male predominance (99.2%) limits the generalizability of findings to female patients with tophaceous gout. This may reflect both the true epidemiology of tophaceous gout and potential referral or health-seeking behavior biases.
5. Short follow-up period: Mean hospitalization of 20.98 days and healing time of 31.43 days preclude assessment of long-term outcomes, including recurrence rates and sustained SUA control.
6. Incomplete data: The retrospective nature resulted in missing data for some variables (e.g., pain scores not recorded for all patients, variable follow-up documentation).
7. Absence of NLR/MLR analysis: This study did not calculate the neutrophil-to-lymphocyte ratio (NLR) or monocyte-to-lymphocyte ratio (MLR), which have been suggested as more specific markers of systemic inflammation and cardiovascular risk. Future prospective studies should incorporate these parameters to provide a more comprehensive assessment of inflammatory status in patients with tophaceous gout.
8. Confounding by comorbidities: Although comorbidities were documented descriptively, they were not adjusted for statistically in comparative analyses due to the study design.

Despite these limitations, this study provides valuable real-world evidence on the surgical management of gouty tophi wounds in a large Chinese cohort, addressing an important gap in the literature. Future prospective, multicenter studies with rigorously designed controls, standardized follow-up protocols, and inclusion of novel inflammatory markers are warranted to refine surgical protocols and validate indications.

## Data Availability

The raw data supporting the conclusions of this article will be made available by the authors, without undue reservation.
